# Odontogenic Fibromyxoma in a Pediatric Patient: A Rare Aggressive Neoplasm With Recurrent and Fatal Outcome

**DOI:** 10.1155/crid/2439166

**Published:** 2025-09-16

**Authors:** Mohd Faizal Abdullah, Fattirah Auni Fauzi

**Affiliations:** ^1^Oral and Maxillofacial Surgery Unit, School of Dental Sciences, Universiti Sains Malaysia, Health Campus, Kota Bharu, Kelantan, Malaysia; ^2^Oral and Maxillofacial Surgery Unit, Hospital Universiti Sains Malaysia, Health Campus, Kota Bharu, Kelantan, Malaysia

**Keywords:** facial deformity, pediatric patient, recurrent odontogenic fibromxyoma

## Abstract

Odontogenic myxomas, a rare intraosseous neoplasm, usually present as a slow rate of bony expansion and are painless in nature, and eventually lead to facial deformity. The tumor is thought to arise from the mesenchyme of the tooth germ and is associated with an impacted tooth. The fact that this tumor is widely distributed throughout the jaws and facial bones suggests its odontogenic neoplasm origin. Furthermore, its histology is similar to that of the developing tooth's stellate reticulum. Fibromyxomas account for a small proportion of all myxomas. We herein report a complex case of recurrent odontogenic fibromyxoma that requires multiple surgical interventions and recurrences that lead to the death of the patient due to disease progression.

## 1. Introduction

Fibromyxomas with a hypocellular matrix and mucoid ground substance of glycosaminoglycans and chondroitin sulfate are responsible for their expeditious growth and reappearance if not resected completely. Local invasion into the cancellous bone beyond radiographically visible margins, no encapsulation in contrast to benign neoplasms, and the presence of mucoid ground substance are the factors responsible for their recurrence [1, 2]. They can present various radiographic patterns, and the most common patterns are unicystic and multilocular. Most multilocular, as well as some unicystic types, exhibit fine intralesional trabeculation in the form of a soap bubble appearance and/or tennis racket pattern [2]. Their sizes vary and can reach more than 4 cm in the case of multilocular myxomas [3]. They do not spread to the regional lymph nodes [4]. These benign lesions are typically confined to the intraoral, most commonly in the posterior parts of the mandible, and extraoral on rare occasions [5, 6]. Radiological, histological, and histochemical examinations are the modalities used to diagnose this lesion [7]. The surgical treatment for fibromyxoma includes enucleation and curettage. Complete resection is strongly advocated to prevent recurrence. A higher rate of recurrence was noted during the first 2 years after surgical resection [4]. Reported recurrence rates for this tumor were between 25% and 43% [8, 9]. Fibromyxoma of the jaws has a higher recurrence rate than any other bony skeleton, leading to a poor outcome [10]. While odontogenic fibromyxoma has been described in the literature, reports in pediatric patients with fatal intracranial extension remain exceptionally rare. This case highlights the unpredictable, aggressive progression of the tumor despite repeated surgical interventions and clear resection margins. It underscores the unique diagnostic challenges in differentiating fibromyxoma from other pediatric jaw lesions, the therapeutic dilemma in balancing surgical morbidity with disease control, and the poor prognosis once soft tissue and intracranial spread occurs. The report is aimed at providing clinical insight into pediatric-specific considerations, management outcomes, and the unpredictable nature of recurrence in this rare tumor. We herein report a case of odontogenic fibromyxoma in a 15-year-old girl with multiple recurrences that led to the death of the patient.

## 2. Case Report

**Figure 1:**
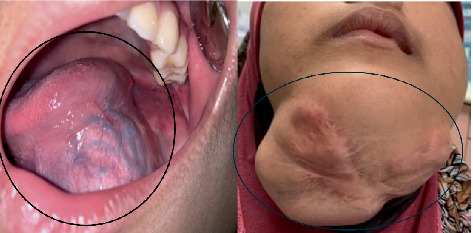
Recurrences at floor of the mouth, tongue, and bilateral submandibular region.

We report a case of odontogenic fibromyxoma in an unfortunate 15-year-old child that underwent multiple surgical interventions for odontogenic fibromyxoma of the head and neck region. The patient was otherwise healthy with no chronic illnesses, no history of facial trauma or radiation exposure, and no relevant family history of neoplastic or maxillofacial conditions. Developmental milestones were normal. Psychosocially, the recurrence and associated facial deformity caused significant school absenteeism, social withdrawal, and emotional distress, requiring counseling support. The patient was referred to the Oral and Maxillofacial Surgery Unit, Hospital Universiti Sains Malaysia, for further management. The diagnosis was established in 2016 with a series of operations done by the referring surgeon. In 2016, the patient underwent a left hemimandibulectomy, and a reconstruction plate was placed. The lesion remained dormant for 2 years; she presented again with swelling over the bilateral submandibular region in 2018, and tumor resection was done by the referring surgeon. However, in 2020, there was another episode of recurrence in the submandibular region, the floor of the mouth, and the tongue, and another tumor resection aiming for a clear margin was performed (Figure 1).

**Figure 2:**
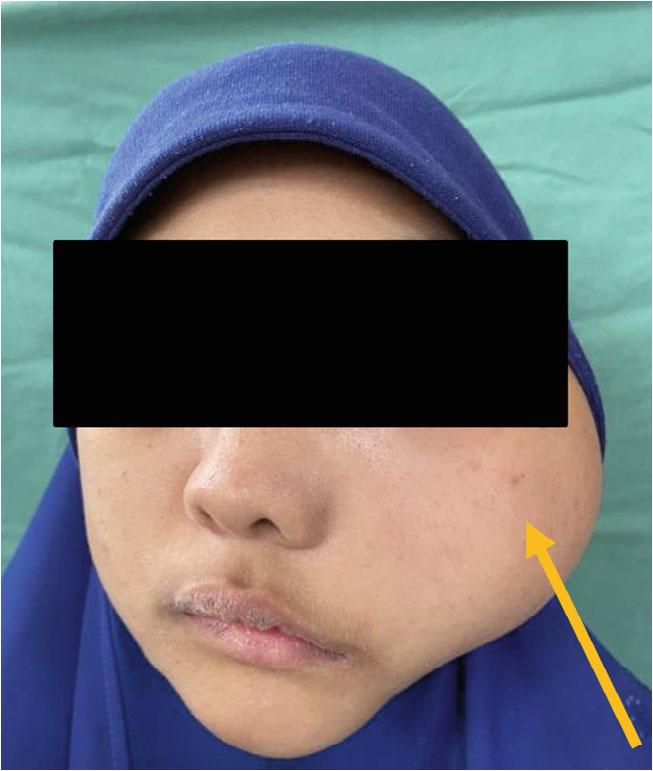
Recurrence at the left face region.

**Figure 3:**
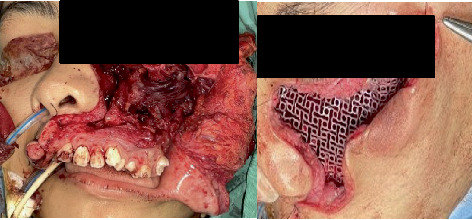
Tumor excision and reconstruction.

In December 2020, she presented again with swelling at the left temporal and the left submandibular region, and wide excision of the tumor was done via the left hemicoronal, left preauricular, and left submandibular region. The tumor, however, recurred at the left maxilla area in August 2021 (Figure 2). Surgical excision of the tumor was done via the left Weber–Ferguson approach, and reconstruction of the left maxilla was done using titanium mesh, and due to tumor impingement of the optic nerve, the patient's left eye function deteriorated (Figure 3).

**Figure 4:**
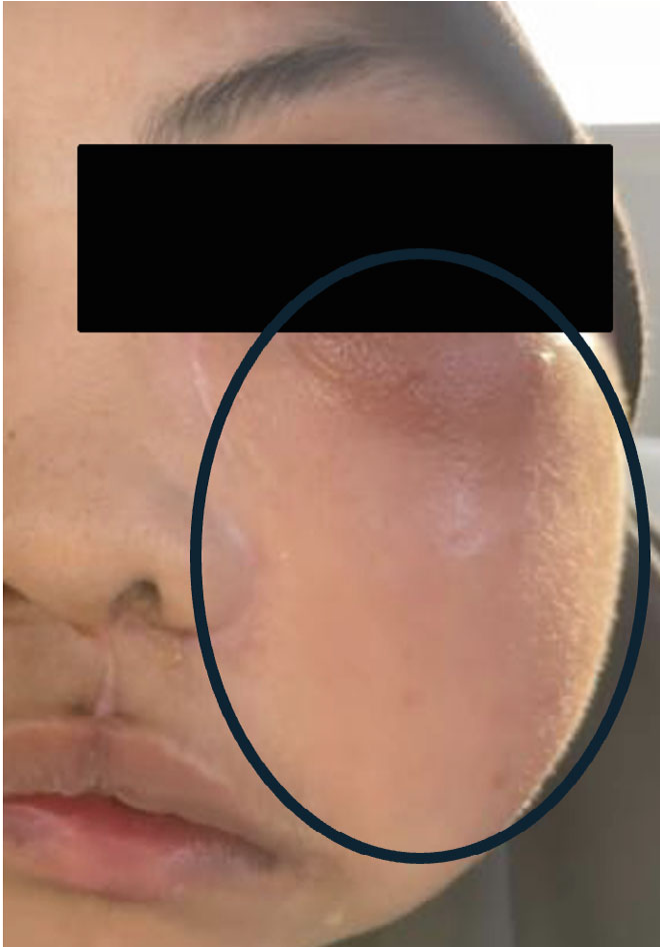
Infected titanium mesh.

Histopathology examinations were consistent with odontogenic fibromyxoma for all the specimens from 2016 to 2021. There were no postoperative complications for the first month, but the patient presented again with an infected titanium plate and signs of recurrence of the tumor at the left maxilla (Figure 4).

**Figure 5:**
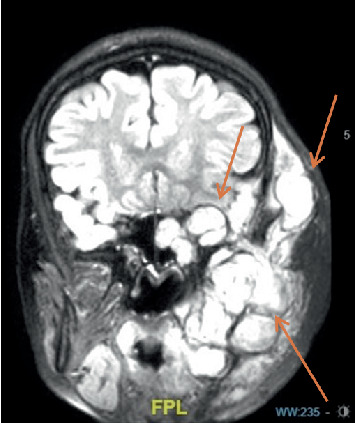
Magnetic resonance imaging (MRI) taken 1 month after titanium mesh removal showed extensive lesion progression with infiltration of the left parapharyngeal, left carotid, and left cranial extension.

Titanium mesh removal was done. A month after the removal of the titanium mesh, tumor progression showed left cranial, carotid, and parapharyngeal extension (Figure 5).  Table 1 shows the simplified version of this patient's chronological event.

The patient's parents refused any further surgical intervention as the frequency of recurrence remained the same. Even though explanations regarding possible complications, such as loss of vision in the left eye, intracranial extension that can lead to neurologic disturbances, and inferior extension leading to airway obstruction, can occur shortly. The patient eventually deteriorated at home due to intracranial extension and was given palliative and best supportive care. The patient passed away in October 2022 due to disease progression. Written informed consent was obtained from the patient's parent for publication of this case report and any accompanying images.

## 3. Discussion

Given the rarity of myxoma as an odontogenic tumor, fibromyxoma is a distinct type of myxoma manifested by higher fibrous and myxoid contents with a predilection for the posterior part of the mandible, that is, angle and ramus [5, 6]. Fibromyxoma can be presented as a well-defined unilocular or multilocular lesion in which the macroscopic appearance shows gelatinous whitish tissue that alters the marrow and deranges jaw architectures. Previous theories contend that the tumor is caused by the degeneration of fibromas, lipomas, and other tissues because of long-standing irritation and degeneration caused by tissue anoxia [11]; however, current research suggests that these tumors develop from dental follicle mesenchyme with fibroblasts responsible for the pathogenesis, but this explanation is insufficient to describe soft tissue myxomas. Dental supporting structures are most likely the source of soft tissue myxomas [12]. Histopathological features of myxoma/fibromyxoma include hypocellularity, the presence of stellate, spindle-shaped cells in a loose myxoid extracellular matrix, and cells with slender, long cytoplasmic prolongations that describe the immature mesenchymal tissue characteristics [13]. A fibromyxoid lesion presented with calcification or ossification, as well as an abundance of collagenous vascularity compared to a myxoma [14] as presented in our case. Current evidence does not support a direct correlation between the degree of fibrous stroma, which is the “fibro-” component, and aggressiveness; instead, the infiltrative, unencapsulated myxoid matrix is considered the main driver of local invasion [15]. Although the exact molecular drivers in this patient were not tested, recent studies suggest that odontogenic myxomas may harbor recurrent copy number alterations, for example, trisomy of Chromosomes 5, 8, and 20, and unique DNA methylation signatures that could underlie their aggressive local invasion [16]. Overexpression of Bcl-2 and increased mast cell infiltration have also been associated with tumor progression and recurrence risk [17]. Aberrant activation of the MAPK/ERK signaling pathway, even without canonical mutations, has been reported in aggressive odontogenic lesions, suggesting a potential role for targeted MEK inhibitor therapy in future cases [18].

Radiological, histological, and histochemical examinations are the modalities used to diagnose myxomas. The radiological examination reveals homogeneous radiolucencies and is described as a “honeycomb,” “soap bubble,” and “tennis racket” appearance [19]. The differential diagnosis for myxoma/fibromyxoma includes ameloblastoma, central haemangioma, fibrous dysplasia, odontogenic cysts, aneurysmal cysts, metastatic tumor, well-differentiated liposarcoma, and desmoplastic fibroma [4]. Recurrence rates for myxomas/fibromyxomas range between 25% and 43% [8, 9] owing to the lesion's sheathless nature, rendering total resection difficult. Complete resection and peripheral osteotomy are the preferred treatments, which lead to a lower recurrence rate [6, 12, 20]. The first surgery in our case was considered successful as the tumor remained dormant for 2 years, but long-term follow-up should be done as the propensity for recurrence of this tumor is high [21]. Despite the extensive resection with a margin of more than 1.5 cm, the extension of the lesion to the soft tissue counterpart, the infiltrative nature of fibromyxomas, including microscopic spread beyond radiographic boundaries, can result in recurrence despite apparently adequate resection [15] renders the difficulty of obtaining a clear margin and subjects the patient to multiple surgical procedures due to recurrences. The decision to stop the surgical intervention, in this case, depends on the surgeon's factor and the patient's factor. In terms of the surgeon's factor, the fast progression of disease and involvement of vital structures such as the eyes, major vessels, and the base of the skull render surgical intervention risky. Alternative treatment such as radiotherapy has limited benefit in odontogenic fibromyxomas [15], but with growing evidence of MAPK/ERK pathway activation in some cases, molecularly targeted agents may emerge as adjunctive treatment for aggressive or unresectable tumors [18]. The patient and her parents decided to stop surgical treatment in our case because of fast recurrence and opted for palliative and supportive care only. This case report highlighted the aggressive nature of fibromyxoma and the dilemma of attending surgeons to obtain a clear margin given the nature of the tumor and its high recurrence rate.

## 4. Conclusion

Odontogenic fibromyxoma is an uncommon, aggressive tumor that most frequently affects the posterior part of the mandible. To ensure optimal patient management, a histopathologic evaluation is mandatory to differentiate this lesion from other pathological entities. Once the tumor invades its soft tissue counterpart, clearance will be difficult, and the tumor can spread very fast to the head and neck region. Pediatric oncologists and maxillofacial surgeons should therefore consider exceptionally aggressive primary surgical approaches with wide, clear margins, accompanied by close, long-term surveillance to detect early recurrence. Moreover, investigating molecular features, such as MAPK/ERK activation or copy number alterations, may help identify high-risk cases and potentially guide novel adjuvant therapies in the future.

## Figures and Tables

**Table 1:** Summary of chronological events.

**Date**	**Event**
2016	Initial presentation with jaw swelling → left hemimandibulectomy and reconstruction plate placed
2018	Recurrence in bilateral submandibular region → tumor resection
2020	Recurrence in submandibular region, floor of mouth, and tongue → resection with clear margin
Dec 2020	Recurrence in left temporal + submandibular region → wide excision via hemicoronal, preauricular, and submandibular approach
Aug 2021	Recurrence in left maxilla → Weber–Ferguson approach excision + titanium mesh reconstruction
Sept 2021	Postop plate infection + recurrence → titanium mesh removal
Oct 2021	Tumor progression with cranial, carotid, and parapharyngeal extension
Oct 2022	Death at home due to intracranial disease progression

## Data Availability

The data that support the findings of this study are available on request from the corresponding author. The data are not publicly available due to privacy or ethical restrictions.
